# *N*-(4-Bromo­phen­yl)urea

**DOI:** 10.1107/S1600536810041735

**Published:** 2010-10-20

**Authors:** Petr Štěpnička, Ivana Císařová

**Affiliations:** a Department of Inorganic Chemistry, Faculty of Science, Charles University in Prague; Hlavova 2030, 12840 Prague 2, Czech Republic

## Abstract

In the title compound, C_7_H_7_BrN_2_O, both the urea moiety [maximum deviation 0.003 (2) Å] and the benzene ring are essentially planar [maximum deviation 0.003 (2) Å] but are rotated with respect to each other by a dihedral angle of 47.8 (1)°. The crystal assembly is stabilized by N—H⋯O hydrogen bonds between all NH protons as conventional hydrogen bond donors and the C=O oxygen as a trifurcated hydrogen-bond acceptor. Both the overall mol­ecular geometry and the crystal packing of the title compound are very similar to those of *N*-phenyl­urea, which is underscored by a practically isostructural relationship between these two urea derivatives.

## Related literature

For the crystal structure of *N*-phenyl­urea, see: Kashino & Haisa (1977 ▶); Bott *et al.* (2000 ▶). For the crystal structure of *N*-(4-tol­yl)urea, see: Ciajolo *et al.* (1982 ▶). For the structure of a mol­ecular 1:1 adduct of *N*-(4-bromo­phen­yl)urea with *N*-(4-bromo­phen­yl)-2-{2-[2-(((4-bromo­phen­yl)carbamo­yl)amino)-2-oxoeth­yl]cyclo­hex-1-en-1-yl}-2-cyano­acetamide, see: Zhang *et al.* (2009 ▶).
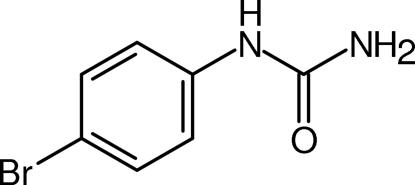


## Experimental

### 

#### Crystal data

C_7_H_7_BrN_2_O
*M* *_r_* = 215.06
Monoclinic, 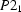

*a* = 4.6033 (2) Å
*b* = 5.3915 (2) Å
*c* = 15.9444 (8) Å
β = 97.994 (3)°
*V* = 391.87 (3) Å^3^
*Z* = 2
Mo *K*α radiation
μ = 5.18 mm^−1^
*T* = 150 K
0.40 × 0.20 × 0.20 mm

#### Data collection

Nonius KappaCCD diffractometer
Absorption correction: gaussian (Coppens, 1970 ▶) *T*_min_ = 0.247, *T*_max_ = 0.475
5026 measured reflections
1771 independent reflections
1704 reflections with *I* > 2σ(*I*)
*R*_int_ = 0.037

#### Refinement

*R*[*F* ^2^ > 2σ(*F* ^2^)] = 0.023
*wR*(*F* ^2^) = 0.056
*S* = 1.05
1771 reflections
103 parameters
1 restraint
H-atom parameters constrained
Δρ_max_ = 0.30 e Å^−3^
Δρ_min_ = −0.30 e Å^−3^
Absolute structure: Flack (1983 ▶), 792 Friedel pairs
Flack parameter: −0.010 (11)

### 

Data collection: *COLLECT* (Nonius, 2000 ▶); cell refinement: *HKL* *SCALEPACK* (Otwinowski & Minor, 1997 ▶); data reduction: *HKL* *DENZO* (Otwinowski & Minor, 1997 ▶) and *SCALEPACK*; program(s) used to solve structure: *SIR97* (Altomare *et al.*, 1999 ▶); program(s) used to refine structure: *SHELXL97* (Sheldrick, 2008 ▶); molecular graphics: *PLATON* (Spek, 2009 ▶); software used to prepare material for publication: *PLATON*.

## Supplementary Material

Crystal structure: contains datablocks I, global. DOI: 10.1107/S1600536810041735/su2219sup1.cif

Structure factors: contains datablocks I. DOI: 10.1107/S1600536810041735/su2219Isup2.hkl

Additional supplementary materials:  crystallographic information; 3D view; checkCIF report

## Figures and Tables

**Table 1:** Hydrogen-bond geometry (Å, °)

*D*—H⋯*A*	*D*—H	H⋯*A*	*D*⋯*A*	*D*—H⋯*A*
N1—H1*N*⋯O1^i^	0.90	2.11	2.904 (3)	146
N2—H2*N*⋯O1^ii^	0.90	2.12	2.979 (3)	158
N2—H3*N*⋯O1^i^	0.93	2.12	2.865 (3)	137

Symmetry codes: (i) 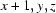; (ii) 
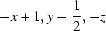
.

**Fig. 1.**
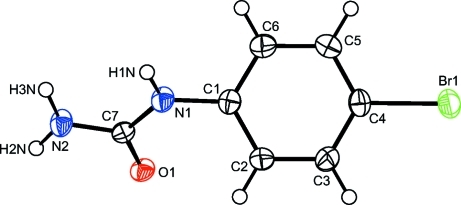
The molecular structure of the title molecule as viewed perpendicularly to the benzene ring. Displacement ellipsoids for the non-H atoms are shown at the 50% probability level. Hydrogen atoms are presented as spheres with an arbitrary radius.

**Fig. 2.**
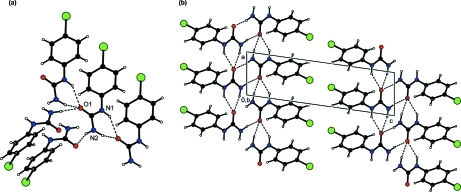
(a) Hydrogen bonds (dashed lines) generated by the molecules of the title compound (see Table 1 for details). (b) Section of the crystal array of the title compound as viewed along the b axis (hydrogen bonds are shown as dashed lines).

## supplementary crystallographic information

### Comment

The title compound crystallized with the symmetry of the monoclinic space group *P*2_1_. Its molecular structure (Fig. 1) compares well to those reported earlier for *N*-phenylurea (Kashino & Haisa 1977; Bott *et al.*, 2000), *N*-(4-tolyl)urea (Ciajolo *et al.*, 1982), and mainly to the structure of *N*-(4-bromophenyl)urea as recently established in the molecular adduct, *N*-(4-bromophenyl)-2-{2-[2-(((4-bromophenyl)carbamoyl)amino)-2-oxoethyl] cyclohex-1-en-1-yl}-2-cyanoacetamide–*N*-(4-bromophenyl)urea (1/1) (Zhang *et al.*, 2009).

The four non-hydrogen atoms constituting the urea moiety in the title molecule are coplanar within 0.003 (2) Å, whilst the atoms forming the benzene ring (C1–C6) depart from their mean plane by only 0.002 (3) Å. The Br1 and N1 atoms are displaced from the latter plane by 0.016 (1) Å and 0.053 (2) Å, respectively. Whereas the bromine atoms binds symmetrically to the aromatic ring (the difference in the C(3/5)—C4—Br1 angles is less than 0.1 °), the C1—N1 bond connecting both functional parts is slightly twisted (*cf.* N1—C1—C2 = 121.5 (2) ° and N1—C1—C6 = 118.8 (3) °). More importantly, the benzene ring and the urea moiety are mutually rotated with a dihedral angle of their mean planes of 47.8 (1) °, which is considerably more than in the afore mentioned adduct (*ca* 16.5 °), but practically identical with the value reported for *N*-phenylurea [46.4 and 47.6 ° depending on the study (Kashino & Haisa, 1977; Bott *et al.*, 2000)].

In the crystal, the individual molecules of *N*-(4-bromophenyl)urea associate predominantly by means of N—H···O hydrogen bonds (Table 1). However, because of the pronounced imbalance in the number of conventional hydrogen bond donors and acceptors, the carbonyl oxygen O1 behaves as a trifurcated hydrogen bond acceptor, interacting with two proximal molecules (Fig. 2a) related by elemental translation along the *a*-axis and a crystallographic twofold screw axis, respectively. This leads to the formation of layers oriented parallel to the *ab* plane (Fig. 2b). Notably, the same array is preserved also for *N*-phenylurea, resulting in similar metrical parameters and the same non-centrosymmetric space group. For *N*-(4-tolyl)urea, on the other hand, similar hydrogen bonded layers related *via* a crystallographic inversion centre, leading to the space group *P*2_1_/c and a doubling of the *c* axis length.

### Experimental

The title compound was obtained from the reaction of sodium cyanate with 4-bromoaniline as described in the literature (Pandeya *et al.*, 2000), and was crystallized from hot 90% ethanol. ^1^H NMR (399.95 MHz, dmso-*d*_6_): δ 5.91 (s, 2H, NH_2_), 7.38 (s, 4H, C_6_H_4_), 8.66 (s, 1H, NH). ^13^C{^1^H} NMR (100.58 MHz, dmso-*d*_6_): δ 112.22 (C*_ipso_* of C_6_H_4_), 119.52 (2CH of C_6_H_4_), 131.18 (2CH of C_6_H_4_), 139.89 (C*_ipso_* of C_6_H_4_), 155.70 (C═O).

### Refinement

The C-bound H atoms were included in calculated positions and refined as riding atoms: C-H = 0.93 Å with U_iso_(H) = 1.2U_eq_(C). The NH and NH_2_ H-atoms were located in a difference electron density map and were refined as riding atoms with *U*_iso_(H) = 1.2*U*_eq_(N).

### Crystal data

**Table:**

C_7_H_7_BrN_2_O	*F*(000) = 212
*M**_r_* = 215.06	*D*_x_ = 1.823 Mg m^−^^3^
Monoclinic, *P*2_1_	Mo *K*α radiation, λ = 0.71073 Å
Hall symbol: P 2yb	Cell parameters from 4819 reflections
*a* = 4.6033 (2) Å	θ = 1.0–27.5°
*b* = 5.3915 (2) Å	µ = 5.18 mm^−^^1^
*c* = 15.9444 (8) Å	*T* = 150 K
β = 97.994 (3)°	Bar, colourless
*V* = 391.87 (3) Å^3^	0.40 × 0.20 × 0.20 mm
*Z* = 2	

### Data collection

**Table:**

Nonius KappaCCD diffractometer	1771 independent reflections
Radiation source: fine-focus sealed tube	1704 reflections with *I* > 2σ(*I*)
horizontally mounted graphite crystal	*R*_int_ = 0.037
Detector resolution: 9.091 pixels mm^-1^	θ_max_ = 27.5°, θ_min_ = 2.6°
ω and φ scans to fill the Ewald sphere	*h* = −5→5
Absorption correction: gaussian (Coppens, 1970)	*k* = −6→6
*T*_min_ = 0.247, *T*_max_ = 0.475	*l* = −20→20
5026 measured reflections	

### Refinement

**Table:**

Refinement on *F*^2^	Secondary atom site location: difference Fourier map
Least-squares matrix: full	Hydrogen site location: difference Fourier map
*R*[*F*^2^ > 2σ(*F*^2^)] = 0.023	H-atom parameters constrained
*wR*(*F*^2^) = 0.056	*w* = 1/[σ^2^(*F*_o_^2^) + (0.0282*P*)^2^ + 0.0852*P*] where *P* = (*F*_o_^2^ + 2*F*_c_^2^)/3
*S* = 1.05	(Δ/σ)_max_ = 0.001
1771 reflections	Δρ_max_ = 0.30 e Å^−^^3^
103 parameters	Δρ_min_ = −0.30 e Å^−^^3^
1 restraint	Absolute structure: Flack (1983), 792 Friedel pairs
Primary atom site location: structure-invariant direct methods	Flack parameter: −0.010 (11)

### Special details

**Table:**

Geometry. All e.s.d.'s (except the e.s.d. in the dihedral angle between two least-squares planes) are estimated using the full covariance matrix. The cell e.s.d.'s are taken into account individually in the estimation of e.s.d.'s in distances, angles and torsion angles; correlations between e.s.d.'s in cell parameters are only used when they are defined by crystal symmetry. An approximate (isotropic) treatment of cell e.s.d.'s is used for estimating e.s.d.'s involving least-squares planes.
Refinement. Refinement of *F*^2^ against all diffractions. The weighted *R*-factor *wR* and goodness of fit *S* are based on *F*^2^, conventional *R*-factors *R* are based on *F*, with *F* set to zero for negative *F*^2^. The threshold expression of *F*^2^ > 2σ(*F*^2^) is used only for calculating *R*-factors(gt) *etc*. and is not relevant to the choice of reflections for refinement. *R*-factors based on *F*^2^ are statistically about twice as large as those based on *F*, and *R*- factors based on all data will be even larger.

### Fractional atomic coordinates and isotropic or equivalent isotropic displacement parameters (Å^2^)

**Table:**

	*x*	*y*	*z*	*U*_iso_*/*U*_eq_	
Br1	0.54956 (5)	1.31775 (8)	0.426555 (13)	0.03508 (9)	
O1	0.4501 (4)	0.5276 (3)	0.08842 (11)	0.0251 (4)	
N1	0.9049 (5)	0.6438 (4)	0.15212 (14)	0.0268 (4)	
H1N	1.1016	0.6285	0.1545	0.039 (9)*	
N2	0.8539 (5)	0.3758 (4)	0.03989 (15)	0.0288 (6)	
H2N	0.7282	0.3042	−0.0015	0.042 (8)*	
H3N	1.0526	0.3368	0.0491	0.046 (8)*	
C1	0.8105 (5)	0.7986 (7)	0.21549 (13)	0.0240 (5)	
C2	0.5960 (6)	0.9794 (5)	0.19521 (16)	0.0291 (6)	
H2	0.5049	0.9971	0.1397	0.035*	
C3	0.5192 (7)	1.1329 (5)	0.25844 (16)	0.0313 (6)	
H3	0.3768	1.2546	0.2454	0.038*	
C4	0.6548 (6)	1.1045 (5)	0.34067 (16)	0.0267 (5)	
C5	0.8670 (6)	0.9267 (5)	0.36161 (17)	0.0316 (6)	
H5	0.9570	0.9095	0.4172	0.038*	
C6	0.9445 (6)	0.7738 (5)	0.29848 (16)	0.0329 (7)	
H6	1.0880	0.6532	0.3119	0.039*	
C7	0.7229 (6)	0.5154 (4)	0.09379 (15)	0.0219 (5)	

### Atomic displacement parameters (Å^2^)

**Table:**

	*U*^11^	*U*^22^	*U*^33^	*U*^12^	*U*^13^	*U*^23^
Br1	0.04673 (17)	0.03249 (14)	0.02688 (12)	0.00234 (17)	0.00808 (9)	−0.00465 (14)
O1	0.0148 (9)	0.0297 (10)	0.0307 (9)	0.0013 (7)	0.0031 (7)	−0.0010 (7)
N1	0.0152 (11)	0.0337 (11)	0.0315 (11)	0.0002 (8)	0.0035 (8)	−0.0076 (9)
N2	0.0182 (10)	0.0353 (16)	0.0335 (11)	−0.0008 (9)	0.0057 (9)	−0.0104 (9)
C1	0.0215 (11)	0.0243 (13)	0.0269 (10)	−0.0023 (14)	0.0063 (8)	−0.0016 (13)
C2	0.0344 (15)	0.0267 (13)	0.0253 (12)	0.0053 (11)	0.0013 (10)	0.0022 (10)
C3	0.0391 (16)	0.0250 (12)	0.0299 (13)	0.0075 (12)	0.0051 (12)	0.0010 (11)
C4	0.0304 (14)	0.0250 (12)	0.0263 (12)	−0.0044 (11)	0.0092 (11)	−0.0033 (10)
C5	0.0319 (15)	0.0357 (12)	0.0256 (12)	0.0017 (12)	−0.0018 (11)	0.0001 (10)
C6	0.0268 (13)	0.037 (2)	0.0330 (12)	0.0058 (12)	−0.0016 (10)	−0.0023 (11)
C7	0.0189 (12)	0.0221 (11)	0.0246 (11)	0.0009 (9)	0.0025 (9)	0.0012 (9)

### Geometric parameters (Å, °)

**Table:**

Br1—C4	1.901 (2)	C1—C2	1.393 (4)
O1—C7	1.249 (3)	C2—C3	1.388 (4)
N1—C7	1.353 (3)	C2—H2	0.9300
N1—C1	1.424 (4)	C3—C4	1.380 (4)
N1—H1N	0.9044	C3—H3	0.9300
N2—C7	1.346 (3)	C4—C5	1.376 (4)
N2—H2N	0.9014	C5—C6	1.386 (4)
N2—H3N	0.9301	C5—H5	0.9300
C1—C6	1.386 (3)	C6—H6	0.9300
			
C7—N1—C1	124.5 (2)	C2—C3—H3	120.1
C7—N1—H1N	120.2	C5—C4—C3	121.3 (2)
C1—N1—H1N	115.2	C5—C4—Br1	119.3 (2)
C7—N2—H2N	114.1	C3—C4—Br1	119.37 (19)
C7—N2—H3N	122.9	C4—C5—C6	118.9 (2)
H2N—N2—H3N	122.4	C4—C5—H5	120.5
C6—C1—C2	119.7 (3)	C6—C5—H5	120.5
C6—C1—N1	118.8 (3)	C5—C6—C1	120.7 (3)
C2—C1—N1	121.4 (2)	C5—C6—H6	119.7
C3—C2—C1	119.5 (2)	C1—C6—H6	119.7
C3—C2—H2	120.2	O1—C7—N2	121.4 (2)
C1—C2—H2	120.2	O1—C7—N1	122.8 (2)
C4—C3—C2	119.8 (2)	N2—C7—N1	115.8 (2)
C4—C3—H3	120.1		
			
C7—N1—C1—C6	132.2 (3)	C3—C4—C5—C6	−0.1 (4)
C7—N1—C1—C2	−50.3 (4)	Br1—C4—C5—C6	−179.3 (2)
C6—C1—C2—C3	0.1 (4)	C4—C5—C6—C1	−0.1 (4)
N1—C1—C2—C3	−177.4 (3)	C2—C1—C6—C5	0.1 (4)
C1—C2—C3—C4	−0.3 (4)	N1—C1—C6—C5	177.6 (3)
C2—C3—C4—C5	0.3 (4)	C1—N1—C7—O1	2.4 (4)
C2—C3—C4—Br1	179.5 (2)	C1—N1—C7—N2	−179.0 (3)

### Hydrogen-bond geometry (Å, °)

**Table:**

*D*—H···*A*	*D*—H	H···*A*	*D*···*A*	*D*—H···*A*
N1—H1N···O1^i^	0.90	2.11	2.904 (3)	146
N2—H2N···O1^ii^	0.90	2.12	2.979 (3)	158
N2—H3N···O1^i^	0.93	2.12	2.865 (3)	137

Symmetry codes: (i) *x*+1, *y*, *z*; (ii) −*x*+1, *y*−1/2, −*z*.
